# Pentraxin-3 dynamics during liver transplantation and association with early allograft dysfunction

**DOI:** 10.3389/frtra.2026.1920891

**Published:** 2026-10-16

**Authors:** Alexandria Page, Michael P. Bokoch, Dieter Adelmann, Rachel Lin, Judith Hellman, Kerstin Kolodzie

**Affiliations:** 1 Department of Anesthesia and Perioperative Care, University of California San Francisco, San Francisco, CA, United States; 2 Department of Epidemiology and Biostatistics, University of California, San Francisco, CA, United States; 3 Philip R. Lee Institute for Health Policy Studies, University of California, San Francisco, CA, United States

**Keywords:** biomarkers, organ preservation, pentraxins, primary graft dysfunction, Vascular Endothelium

## Abstract

**Introduction:**

Liver transplantation involves significant Ischemia-Reperfusion Injury (IRI). Recipients frequently exhibit features of endothelial dysfunction, such as inflammation and coagulopathy, which may be associated with adverse outcomes, including early allograft dysfunction (EAD). Pentraxin-3 (PTX-3) is involved in vascular endothelial inflammation and dysfunction, and is synthesized by endothelial cells and leukocytes. In this retrospective cohort study, we hypothesized that PTX-3 levels are associated with EAD.

**Methods:**

PTX-3 levels were quantified in plasma samples collected during liver transplantation at the following time points: start of surgery (T1); end of liver dissection (T2); after reperfusion (T3 at 15 minutes; T4 60-120 minutes). Control samples from healthy living donors were collected at the start and end of surgery.

**Results:**

Plasma PTX-3 levels were higher during liver transplantation than in healthy controls at T1 (median 1.72 ng/mL, IQR 1.13-2.44 vs. 0.57 ng/mL, IQR 0.44-0.74), *p*<0.01, and increased over time in serial samples collected at T1, T3, and T4 (*n* = 50), *p*<0.001. In those who developed EAD, delta-PTX-3 after reperfusion (T3 to T4) was greater (median 12.3 ng/mL, IQR 6.65-18.7, *n*=17) compared to those without EAD (median 6.59 ng/mL, IQR 2.87-13.2, *n*=34), *p*=0.048, but there was no significant difference in final T4 PTX-3 levels. Delta-PTX-3 after reperfusion was greater in recipients of grafts that received normothermic machine perfusion (NMP) (median 12.7 ng/mL, IQR: 7.90-16.3, *n*=19), vs. Living Donor Liver Transplants (median 5.79ng/mL, IQR 2.60-10.2, *n*=24), *p*=0.02.

**Discussion:**

Circulating PTX-3 levels increased during liver transplantation, with the highest levels after reperfusion. This suggests IRI triggered an increase in PTX-3. After reperfusion, delta-PTX-3, but not final PTX-3, was associated with an increased incidence of EAD and was greatest in recipients of grafts after NMP, suggesting NMP may involve earlier activation of pathways downstream of IRI.

## 1 Introduction

Liver transplantation is a complex surgical intervention that is characterized by ischemia-reperfusion injury (IRI) of the liver graft, and the severity of IRI is associated with EAD and graft failure (1). Endothelial dysfunction downstream of liver IRI is postulated to promote post-transplantation complications, including EAD (2–5). Inflammation and dysregulated coagulation are key features of endothelial dysfunction and are associated with adverse outcomes after liver transplant (6–8). To examine this relationship, we studied Pentraxin-3 (PTX-3) an acute phase protein that modulates endothelial function, in patients with cirrhosis undergoing liver transplantation and its association with EAD.

PTX-3, a member of the pentraxin superfamily that is a homolog of C-reactive protein, is produced by endothelial cells and leukocytes, and participates in innate immune responses, inflammation, and tissue remodeling (9, 10). PTX-3 plays roles in three key endothelial functions: mediating the innate immune response; modulating *P*-selectin-mediated leukocyte recruitment; and influencing coagulation (10–13). PTX-3 rises rapidly in response to acute stimuli, such as sepsis and myocardial infarction. In ICU admissions for meningococcal sepsis, it peaked within hours and differentiated between patients presenting with and without shock (14). Although physiological reference levels have not yet been validated, one study reported concentrations measured at an annual check-up of 2.21 ng/mL (2.05–2.19) in healthy women and 1.87 ng/mL (1.81–1.94) in healthy men (15). In contrast, PTX-3 levels in severe disease states are frequently reported as 10-fold higher. For example, PTX-3 levels of 26.9 ng/mL have been reported as an optimal cut-off for predicting 28-day mortality in sepsis (16). However, exogenous administration or overexpression of PTX-3 has been reported to increase survival in mice with endotoxin-induced shock, suggesting a protective role in acute inflammation (17, 18).

The role of Pentraxin-3 in mediating IRI has been investigated in other organs. For instance, PTX-3 has been studied in mouse models of renal IRI, with evidence of both protective and deleterious roles (19). Although rising PTX-3 levels have been demonstrated in human and mouse liver biopsies during hepatic IRI, the relationship between circulating PTX-3 and clinical outcomes in liver transplant remains underexplored (20). Our study, which focuses on circulating PTX-3 levels, complements other studies that focus on tissue levels, as intraoperative circulating PTX-3 levels may differ from liver biopsy mRNA expression, reflecting disparate sources and actions. Biomarkers of different elements of endothelial dysfunction such as the glycocalyx component syndecan-1, have already been reported to predict EAD (5).

EAD, the earliest standardized outcome measure of liver transplantation, refers to dysfunction of the engrafted organ in the immediate period following transplant (8). EAD is reported to occur in approximately 25% of cases and is often suggested as an intermediate outcome measure along the path from IRI to graft loss (8, 21). Acute Kidney Injury (AKI) after liver transplant is another clinical outcome that is associated with endothelial dysfunction and IRI (22, 23).

New graft preservation methods, such as Normothermic Machine Perfusion (NMP), have been shown to reduce the risk of EAD, increase the use of marginal grafts, and improve graft and patient survival compared to Static Cold Storage (SCS) (24, 25). NMP reduces but does not eliminate ischemic injury, and grafts undergoing both preservation types were included in this study (26).

We hypothesized that liver transplantation modulates circulating PTX-3 levels, and higher PTX-3 levels are associated with EAD. To examine this association, we measured PTX-3, throughout liver transplant surgery and during donor liver resection, expecting PTX-3 to increase during transplant surgery and to be associated with EAD.

## 2 Materials and methods

We performed a retrospective cohort study of adult liver transplant recipients and healthy living donors at a single academic transplant center, recruited to a Biobank between 5/1/21 and 8/10/25. The biobank protocol was approved by the University of California, San Francisco, Institutional Review Board (IRB #20-30179), and all subjects signed written informed consent. All research was conducted in accordance with both the Declaration of Helsinki and the Declaration of Istanbul. Exclusion criteria included a lack of decision-making capacity, preoperative intubation, age less than 18 years, and a language other than English, Spanish, Mandarin, or Cantonese.

Citrated plasma samples were collected during liver transplant surgery at the start of surgery (T1), the end of dissection (T2), and after reperfusion (Time points: T3 at 15 min; T4 60–120 min) as demonstrated in Figure 1. In the control cohort of healthy living donors, plasma samples were collected at the start and end of donor hepatectomy. All plasma samples were centrifuged at 1850 RCF for 10 min, plasma was aliquoted and stored at −80 °C until analysis. PTX-3 was measured using an enzyme-linked immunosorbent assay (ELISA) according to the manufacturer's instructions (Human Pentraxin 3/TSG-14 Quantikine ELISA Kit, R&D Systems, Minneapolis, MN, USA). Samples were diluted in assay buffer to fall within the assay's linear range. Exposure variable PTX-3 was examined in both absolute and delta formats. Absolute PTX-3 values are reported in ng/mL, and the delta-PTX-3 concentration (ng/mL) was calculated for patients with paired samples (T1, T3, T4) for T4 -T1 and T4-T3.

**Figure 1:**
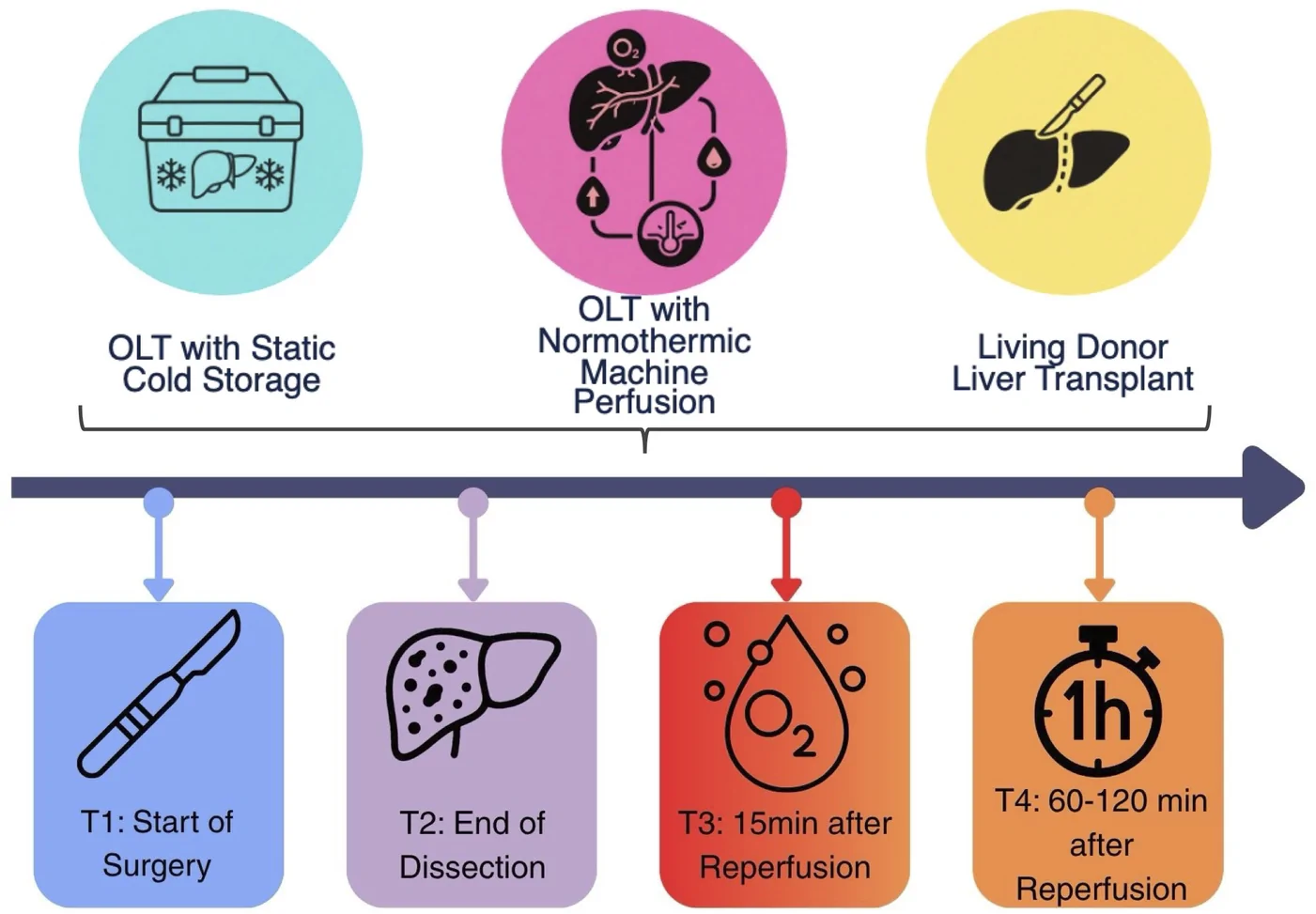
Timepoints for sample collection and types.

Patient and donor data were obtained from the Transplant Outcomes Anesthesia Database, a data warehouse that includes clinical and demographic data (27). Data quality was ensured through manual validation of a random subset and the identification and inspection of the five highest and lowest values for MEAF (Model for Early Allograft Function Scoring), MELD-Na, and components of EAD and AKI (28, 29). The primary outcome, EAD, was defined by the Olthoff criteria, and its components were examined (8). Secondary outcomes included MEAF score, calculated according to the published formula, and AKI, defined according to the International Club of Ascites criteria and reported if it occurred within 48 h of the end of surgery (28, 29). Patients with preoperative dialysis were excluded from AKI analysis. MELD-Na scores were used throughout for consistency over time, as MELD 3.0 was implemented mid-study and was therefore recorded for only a subset of patients (30, 31). The donor risk index was defined according to the model published by the Scientific Registry of Transplant Recipients, and missing race values were imputed using the modal response (32). To compare different methods for graft preservation, we used a three-level variable to describe graft preservation type: recipients from LDLT (Living Donor Liver Transplant); recipients from deceased donors with NMP of graft; recipients from deceased donors with SCS of graft (Reference level). We conducted an exploratory analysis of PTX-3 changes during surgery stratified by graft preservation type. Other intraoperative variables examined included the infused volume of crystalloids, the infusion of at least 1 unit of red blood cells, cold ischemia time (time from donor aortic cross-clamp to out of ice), and the implantation warm ischemic time (minutes from removing the graft from ice to reperfusion through the portal vein). Patients were followed up until retransplant or death. Follow-up was censored if it was fewer than 90 days after transplant.

### 2.1 Statistical analysis

Continuous variables were presented as median and IQR and compared between groups using a 2-tailed Mann–Whitney U test for unpaired data or a paired Wilcoxon signed-rank test for paired data. The distribution of variables across comparative groups was examined, and the equality-of-medians test was used when the groups' distribution differed visually. Categorical variables were reported as counts and proportions and compared using the Chi-squared or Fisher's exact test. Associations between two continuous variables were examined using Spearman's correlation or univariable regression. Three-level comparisons of PTX-3 by graft preservation type were analyzed using the Kruskal–Wallis test, with subsequent pairwise Wilcoxon tests with Bonferroni correction. Tests used available subsets of results, and the number of samples included per comparison is reported. PTX-3's association with MEAF was examined using linear regression. Area under the Receiver-Operating Characteristic (AUROC) was calculated for both final and delta-PTX-3 levels for EAD, and 95% confidence intervals were obtained by stratified bootstrapped resampling (2000 replicates). *P*-values <0.05 were considered statistically significant. Data analysis was performed in GraphPad Prism (version 10.6.0) and R version 4.5.1 [R Core Team (2025); R Foundation for Statistical Computing, Vienna, Austria] with the RStudio interface (Version 2025.09.2 + 418).

## 3 Results

PTX-3 levels were quantified in 76 liver transplant recipient patients and 7 living donor patients. Baseline characteristics and perioperative variables are shown in Table 1. There were 46 LDLT recipients and 30 recipients of livers from deceased donors, 22 of whom received livers that underwent NMP compared to 8 with SCS. There were 8 livers from donation after circulatory death (DCD), 6 of which underwent NMP and 2 had SCS. PTX-3 concentrations for all transplant recipients at time points T1 to T4 are shown in Figure 2. Seventy-five transplant recipients had T1 samples, 51 had T4 samples, and all had more than one sample. Fifty patients had sets of samples at all the following three time points: the start of surgery (T1), 15 min after reperfusion (T3), and 60–120 min after reperfusion (T4).

**Table 1:** All liver transplant patients demographics and perioperative characteristics.

Variable	Overall (*n* = 76)^a^
Age (years)	60 (51, 66)
Sex (% Female)	29 (38%)
BMI (kg/m^2^)	27.1 (24.5, 31.5)
Race	
Non-White	28 (37%)
Unknown/Missing/Other	1 (1.3%)
White	47 (62%)
Diagnosis	
Alcoholic	9 (12%)
Hepatitis B	4 (5.3%)
Hepatitis C	4 (5.3%)
MASH	15 (20%)
Other	34 (45%)
Primary Sclerosing Cholangitis	10 (13%)
HCC Diagnosis (%)	26 (34%)
Case Type (%)	
Liver Transplant	71 (93%)
Liver/Kidney	5 (6.6%)
MELD-Na Score	19 (14, 23)
Preoperative Dialysis (%)	7 (9.2%)
DCD (%)	8 (10.5%)
Graft Preservation Type (%)	
Living Donor (LDLT)	46 (61%)
Deceased Donor (NMP)	22 (29%)
Deceased Donor (SCS)	8 (11%)
IVC Management (%)	
Caval Replacement	6 (8.0%)
Piggyback	69 (92%)
Missing	1
Estimated Blood Loss (l)	1.65 (1.00, 4.25)
Received red cell transfusion (%)	60 (79%)
Intravenous crystalloids (l)	2.00 (1.28, 3.00)
EAD (%)	19 (25%)
AKI (%)	46 (67%)
MEAF	4.47 (3.43, 5.64)

AKI (Acute Kidney Injury); BMI (Body Mass Index); DCD (Donation after circulatory death); HCC (Hepatocellular Carcinoma); EAD (Early Allograft Dysfunction); LDLT (Living Donor Liver Transplant); MASH (Metabolic-dysfunction Associated Steatohepatitis); MEAF (Model of Early Allograft Dysfunction); MELD-Na (Model for End-Stage Liver Disease-Sodium); NMP (Normothermic Machine Perfusion); PTX-3 (Pentraxin-3); SCS (Static Cold Storage).

a Median (IQR); *n* (%).

**Figure 2:**
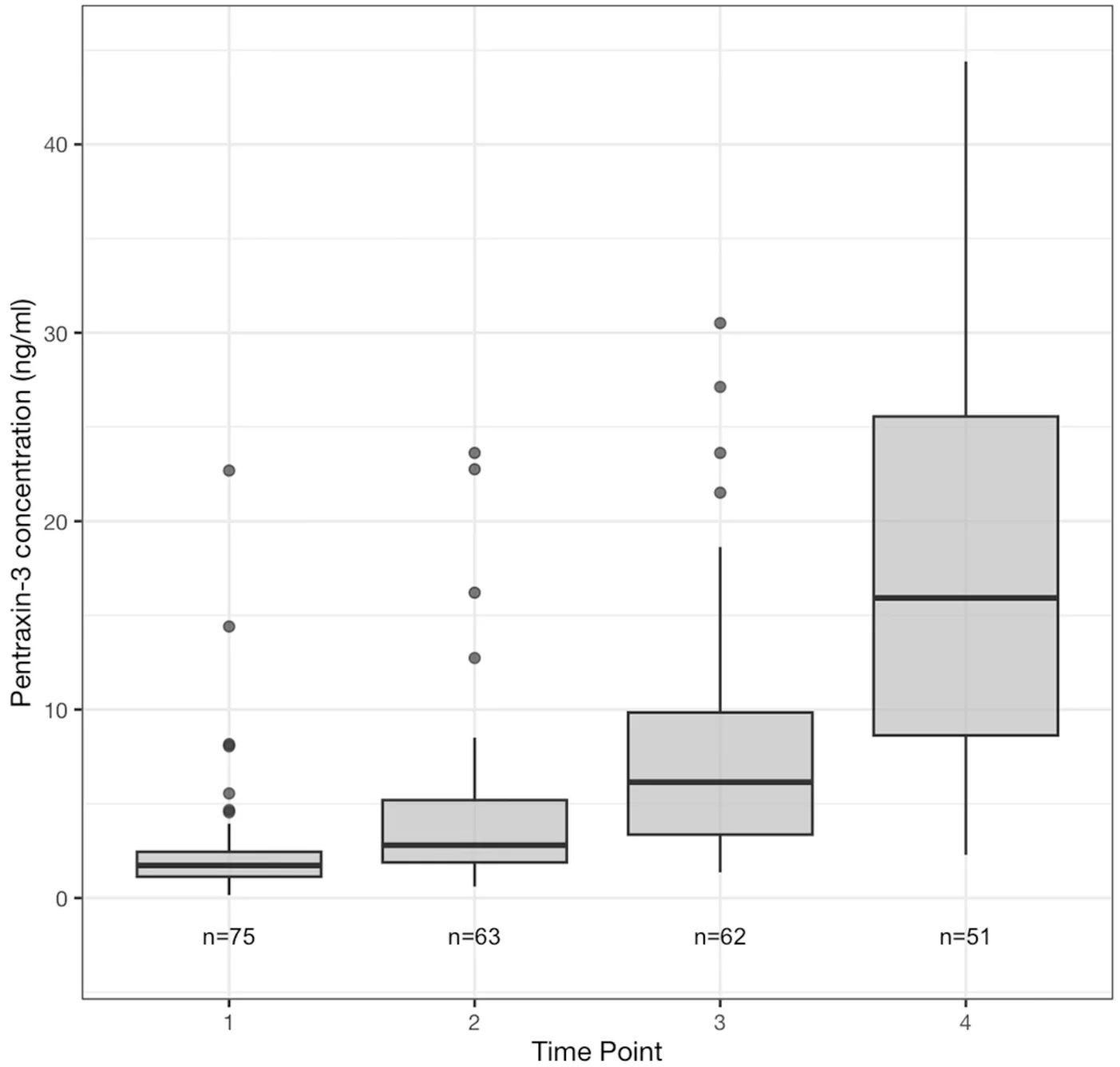
Plasma pentraxin-3 concentration by timepoint for all liver transplant recipients (*n* = 76).

### 3.1 Association of preoperative characteristics and baseline PTX-3

The initial median PTX-3 concentration in all liver transplant recipients (T1) was greater than in healthy living donors at the start of surgery (1.72 ng/mL, IQR: 1.13–2.44, *n* = 75 vs. 0.57 ng/mL, IQR: 0.44–0.74, *n* = 7), *p* = 0.002. In transplant recipients, there was a significant correlation between T1 PTX-3 concentration and the preoperative MELD-Na score (Spearman's Rho = 0.25, *p* < 0.05), as shown in Supplementary Figure S1. There was no difference in the median T1 PTX-3 concentrations in patients with versus without a diagnosis of Hepatocellular carcinoma (HCC) (1.73 ng/mL, IQR:1.28–2.42; *n* = 25) vs. (1.73 ng/mL, IQR: 1.12–2.69, *n* = 50) *p* = 0.85.

The median T1 PTX-3 concentration differed by sex of the liver transplant recipient (Males 1.82 ng/mL, IQR 1.47–2.74, *n* = 46; Females 1.22 ng/mL, IQR 0.69–1.96, *n* = 29, *p* < 0.01). Seven of the 30 recipients of deceased donors were female, compared to 22 of 46 of living donors (23.3% vs. 47.8%, *p* = 0.07), and MELD-Na scores did not differ by sex (*p* = 0.1).

### 3.2 PTX-3 change during surgery

In 50 liver transplant recipients with paired samples, median PTX-3 concentration increased from T1, the start of surgery (1.74 ng/mL, IQR 1.15–2.95), to T3, 15 min after reperfusion (6.70 ng/mL, IQR 3.36–11.4), and to T4, 60–120 min after reperfusion (15.2 ng/mL, IQR 8.38–25.1), *p* < 0.001. The largest increase in PTX-3 occurred between T3 and T4 (median delta-PTX-3 T3 to T4: 7.22 ng/mL, IQR 3.61–13.8), and the T3 to T4 increase was more than double the increase from T1 to T3 (median delta-PTX-3 T1 to T3: 3.36 ng/mL, IQR 1.31–7.87), *p* < 0.001.

Final concentration (T4) in all transplant recipients (15.9 ng/mL; IQR 8.63–25.6, *n* = 51) was greater than at the end of surgery in healthy living donors (1.78 ng/mL; IQR 0.93–3.43, *n* = 7), *p* < 0.001. In the 7 control donor patients, median PTX-3 concentration increased from the start of surgery (0.57 ng/mL, IQR: 0.44–0.74) to the end of surgery (1.78 ng/mL, IQR: 0.93–3.43), *p* = 0.03.

The volume of crystalloid fluids given intraoperatively positively correlated with T4 PTX-3 concentration (*p* = 0.02). The final T4 PTX-3 concentration did not differ between patients who received at least 1 unit of packed red cells intraoperatively and those who did not (*p* = 0.94). Median PTX-3 concentration differed by sex at T4, 60–120 min after reperfusion (Males: 21.2 ng/mL, IQR 12.2–26.7, *n* = 35), compared to (Females: 7.98 ng/mL, IQR 5.79–12.9, *n* = 16), *p* < 0.01.

### 3.3 Association of PTX-3 with graft preservation method

Patient characteristics of the 51 patients who had T4 PTX-3 samples are described in Table 2 by graft preservation type, including PTX-3 levels at T4 and delta PTX-3 (T3 to T4). There was no difference in PTX-3 concentration between graft preservation type at T4 (*p* = 0.1).

**Table 2:** Liver transplant patient demographics and perioperative characteristics with samples at T4 by graft preservation type.

Variable	Graft Preservation Type (Donor Status and Preservation Use)				*p*-value^b^
	Overall (*n* = 51)^a^	Deceased Donor (SCS) *n* = 8^a^	Deceased Donor (NMP) *n* = 19^a^	Living Donor (LDLT) *n* = 24^a^	
Age (years)	60 (50, 66)	63 (57, 68)	61 (55, 67)	57 (41, 63)	0.14
Sex (% Female)	16 (31%)	3 (38%)	4 (21%)	9 (38%)	0.5
BMI (kg/m^2^)	27.4 (24.6, 31.9)	26.9 (19.6, 33.7)	27.4 (25.3, 30.5)	27.9 (23.7, 33.1)	>0.9
Race					0.6
White	34 (67%)	4 (50%)	13 (68%)	17 (71%)	
Non-White	17 (33%)	4 (50%)	6 (32%)	7 (29%)	
Diagnosis					0.5
Alcoholic	7 (14%)	0 (0%)	3 (16%)	4 (17%)	
Hepatitis B	3 (5.9%)	1 (13%)	2 (11%)	0 (0%)	
Hepatitis C	3 (5.9%)	1 (13%)	1 (5.3%)	1 (4.2%)	
MASH	8 (16%)	2 (25%)	4 (21%)	2 (8.3%)	
Other	23 (45%)	3 (38%)	8 (42%)	12 (50%)	
Primary Sclerosing Cholangitis	7 (14%)	1 (13%)	1 (5.3%)	5 (21%)	
HCC Diagnosis (%)	21 (41%)	5 (63%)	11 (58%)	5 (21%)	0.023
Case Type (%)					0.042
Liver Transplant	47 (92%)	6 (75%)	17 (89%)	24 (100%)	
Liver/Kidney	4 (7.8%)	2 (25%)	2 (11%)	0 (0%)	
MELD-Na Score	18 (13, 24)	26 (12, 32)	19 (14, 31)	17 (13, 20)	0.6
Preoperative Dialysis (%)	6 (12%)	2 (25%)	4 (21%)	0 (0%)	0.02
DCD Donor (%)	6 (12%)	2 (25%)	4 (21%)	0 (0%)	0.02
Donor Risk Index	1.64 (1.45, 1.83)	1.45 (1.25, 1.75)	1.67 (1.54, 1.86)	NA	0.15
Missing	25	0	1	24	
WIT (minutes)	32 (28, 37)	31 (28, 35)	31 (26, 37)	33 (28, 43)	0.6
Missing	3	0	0	3	
CIT (hours)	3.25 (1.80, 6.50)	2.70 (1.70, 3.30)	NA	7.1 (6.6, 8.5)	
Missing	23	4	19	0	
IVC Management (%)					0.5
Caval Replacement	4 (8.0%)	1 (13%)	2 (11%)	1 (4.3%)	
Piggyback	46 (92%)	7 (88%)	17 (89%)	22 (96%)	
Missing	1	0	0	1	
Estimated Blood Loss (l)	2.00 (1.00, 4.50)	1.90 (1.35, 4.60)	2.80 (1.50, 6.00)	1.55 (1.00, 3.38)	0.2
Received Red Cell Transfusion (%)	41 (80%)	19 (79%)	15 (79%)	7 (88%)	>0.9
Intravenous crystalloids (l)	2.00 (1.40, 3.00)	1.60 (1.50, 2.75)	2.00 (1.20, 2.50)	2.23 (1.45, 3.00)	0.4
PTX-3 concentration: T4 (ng/mL)	15.9 (8.63, 25.6)	8.63 (6.71, 13.3)	21.2 (12.3, 24.3)	13.7 (7.42, 27.2)	0.1
Delta-PTX-3: T3 to T4 (ng/mL)	7.56 (3.64, 14.0)	5.44 (2.65, 9.81)	12.7 (7.90, 16.3)	5.79 (2.60, 10.2)	0.02

BMI (Body Mass Index); CIT (Cold Ischemia Time); DCD (Donation after circulatory death); HCC (Hepatocellular Carcinoma); LDLT (Living Donor Liver Transplant); MASH (Metabolic-dysfunction Associated Steatohepatitis); MELD-Na (Model for End-Stage Liver Disease-Sodium); NMP (Normothermic Machine Perfusion); PTX-3 (Pentraxin-3); SCS (Static Cold Storage); WIT (Warm Ischemic Time).

a Median (IQR); *n* (%).

b Kruskal–Wallis rank sum test; Fisher's Exact Test for Count Data with simulated *p*-value (based on 2,000 replicates); Fisher's exact test; Mood's median test.

Delta-PTX-3 (T3 to T4) concentration differed by graft preservation types (*p* = 0.02). Corrected pairwise comparisons indicated that recipients of livers from deceased donors with grafts that had NMP (12.7 ng/mL, IQR: 7.9–16.3, *n* = 19) had higher levels than in LDLT (5.79 ng/mL, IQR 2.60–10.2, *n* = 24), *p* = 0.02. There was no difference between recipients of livers from deceased donors with grafts that had NMP compared to SCS (5.44 ng/mL, IQR 2.65–9.81, *n* = 8), *p* = 0.20, nor between LDLT and recipients of livers from deceased donors with grafts that had SCS, *p* = 1.0. Warm ischemic time was not associated with PTX-3 concentration at T4 (*p* = 0.19). PTX-3 concentrations by graft preservation method at all time points measured are shown in Figure 3.

**Figure 3:**
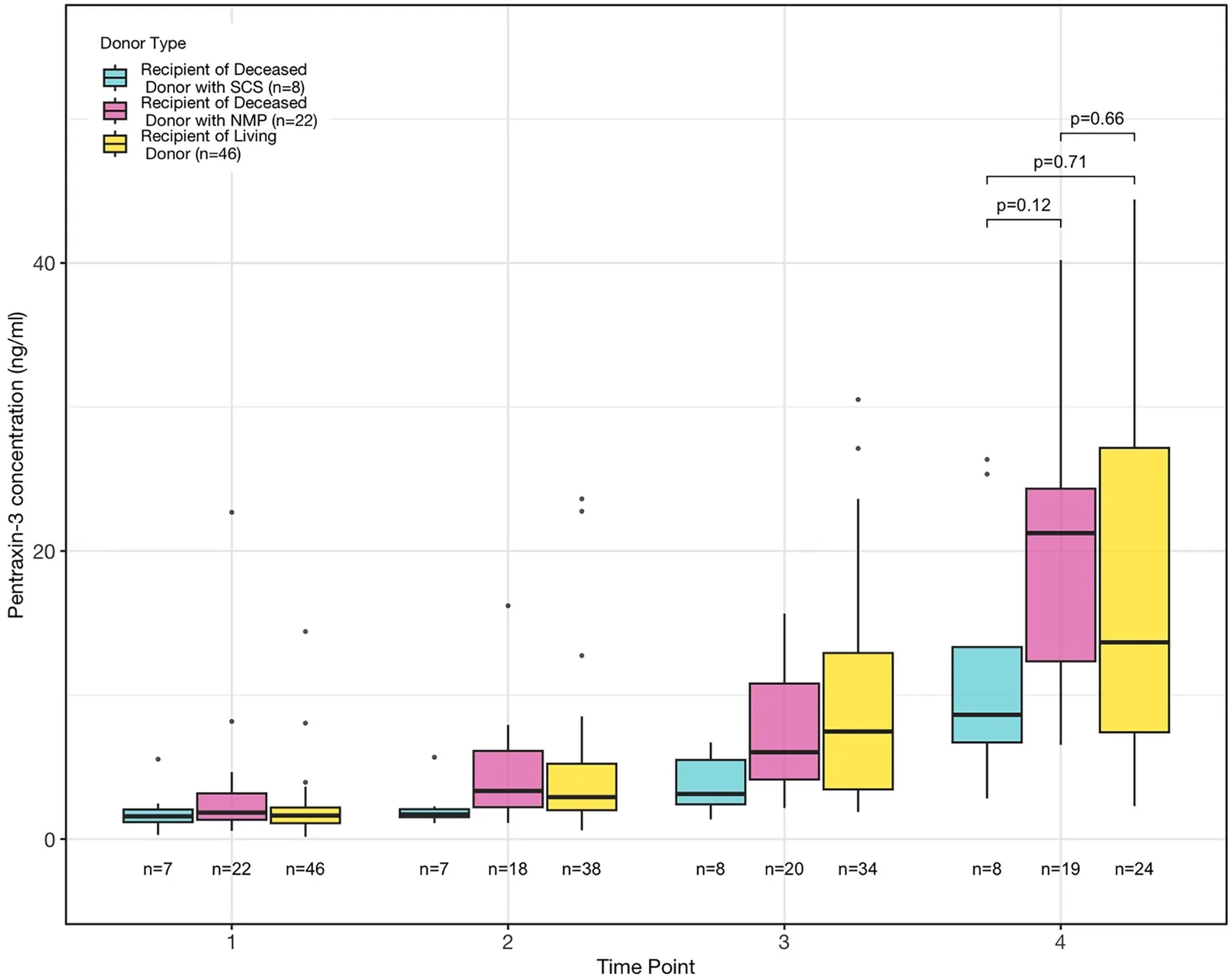
Plasma pentraxin-3 (ng/mL) change over time for all liver transplant reciepients (*n* = 76) by graft preservation type (donor status and preservation use). Pairwise comparisons by the Wilcoxon-Rank Sum test, adjusted *p*-values are reported with Bonferroni correction.

In those with T4, the 6 recipients of grafts from DCD had median T4 PTX-3 of 18.6 ng/mL (IQR: 9.86–25.1) compared to 17.8 ng/mL (IQR: 9.18–22.9) in the 21 with grafts following donation after brain death (DBD), *p* = 0.89. In those with T4, the median delta-PTX-3 was 12.5 ng/mL (IQR 6.24–17.9) in recipients of grafts from DCD, and 9.53 ng/mL (IQR 6.65–15.4) in recipients of grafts from DBD, *p* = 0.67. In those with T4 and grafts that underwent NMP, median T4 was 18.9 ng/mL (IQR 13.9–25.6) in the 4 who received grafts from DCD compared with 21.9 ng/mL (IQR 12.3–24.3) in the 15 who received grafts after DBD, *p* = 0.33, median test was used due to differing distributions. Median delta-PTX-3 was 12.5 ng/mL (IQR 10.1–17.3) in recipients of grafts from DCD that underwent NMP compared with 13.1 ng/mL (IQR 7.90–16.3) in recipients of grafts from DBD that underwent NMP, *p* = 1.0.

In those with T4, and recipients of grafts that underwent SCS, median T4 was 8.63 ng/mL (IQR: 5.57–9.29) in the 6 who received grafts from DBD compared with 7.82 ng/mL and 26.4 ng/mL in the 2 who received grafts from DCD and underwent SCS. In those who had T4, delta-PTX-3 was 4.23 ng/mL and 19.6 ng/mL in the 2 who received grafts from DCD that underwent SCS, and was 4.75 ng/mL (IQR 2.23–6.69) in the 6 recipients of grafts from DBD that underwent SCS.

### 3.4 Clinical outcomes

EAD was observed in 17 of 51 patients with final PTX-3 samples (33%), and their characteristics are described in Table 3 by EAD status. In patients who developed EAD, there was no significant difference in the median T4 PTX-3 concentration (20.1 ng/mL, IQR 13.0–25.3, *n* = 17) compared to those who did not develop EAD (12.4 ng/mL, IQR 6.85–25.1, *n* = 34), *p* = 0.12, in those with final PTX-3 measured. EAD was examined by median test due to the presence of right skew in the group without EAD, which was not present in those with EAD. The median delta-PTX-3 T3 to T4 concentration, 15 to 60–120 min after reperfusion, was greater in patients who developed EAD (12.3 ng/mL, IQR 6.65–18.7, *n* = 17) compared to those who did not (6.59 ng/mL, IQR 2.87–13.2, *n* = 34), *p* = 0.048. The ROC curve for EAD gave an AUC for T4 PTX-3 of 0.63 (0.48–0.78) and for delta-PTX-3 after reperfusion AUC was 0.67 (0.51–0.82) as demonstrated in Figure 4. The maximum ALT or AST within 7 days of surgery was not correlated with T4 PTX-3 concentration (*p* = 0.25) but was correlated with the delta-PTX-3 (T3 to T4) in patients with paired samples (*p* < 0.01). In those with final PTX-3 samples who were recipients of grafts from DCD, all 6 had EAD. Of these, 2 received organs preserved with SCS with a median T4 PTX-3 17.1 ng/mL and delta-PTX-3 of 11.9 ng/mL, compared to 4 grafts that underwent NMP with a median T4 PTX-3 of 18.6 ng/mL and a delta-PTX-3 of 12.5 ng/mL.

**Table 3:** Liver transplant patient demographics and perioperative characteristics with samples at T4 by early allograft dysfunction.

Variable	EAD			*p*-value^b^
	Overall (*n* = 51)^a^	No *n* = 34^a^	Yes *n* = 17^a^	
Age (years)	60 (50, 66)	60 (45, 69)	59 (55, 62)	0.6
Sex (% Female)	16 (31%)	13 (38%)	3 (18%)	0.2
BMI (kg/m^2^)	27.4 (24.6, 31.9)	28.9 (25.1, 32.5)	25.7 (22.0, 29.7)	0.3
Race				>0.9
White	34 (67%)	23 (68%)	11 (65%)	
Non-White	17 (33%)	11 (32%)	6 (35%)	
Diagnosis				0.4
Alcoholic	7 (14%)	5 (15%)	2 (12%)	
Hepatitis B	3 (5.9%)	1 (2.9%)	2 (12%)	
Hepatitis C	3 (5.9%)	3 (8.8%)	0 (0%)	
MASH	8 (16%)	5 (15%)	3 (18%)	
Other	23 (45%)	17 (50%)	6 (35%)	
Primary Sclerosing Cholangitis	7 (14%)	3 (8.8%)	4 (24%)	
HCC Diagnosis (%)	21 (41%)	13 (38%)	8 (47%)	0.8
Case Type (%)				>0.9
Liver Transplant	47 (92%)	31 (91%)	16 (94%)	
Liver/Kidney	4 (7.8%)	3 (8.8%)	1 (5.9%)	
MELD-Na Score	18 (13, 24)	17 (13, 21)	23 (14, 27)	0.10
Preoperative Dialysis (%)	6 (12%)	4 (12%)	2 (12%)	>0.9
Graft Preservation Type (%)				0.012
Deceased Donor (SCS)	8 (16%)	4 (12%)	4 (24%)	0.42
Deceased Donor (NMP)	19 (37%)	9 (26%)	10 (59%)	0.07
Living Donor	24 (47%)	21 (62%)	3 (18%)	0.01
DCD Donor (%)	6 (12%)	0 (0%)	6 (35%)	<0.001
WIT (minutes)	32 (28, 37)	30 (28, 37)	35 (31, 37)	0.088
Missing	3	3	0	
Donor Risk Index	1.64 (1.45, 1.83)	1.63 (1.49, 1.72)	1.68 (1.34, 2.22)	0.6
Missing	25	22	3	
IVC Management (%)				0.11
Full	4 (8.0%)	1 (3.0%)	3 (18%)	
Piggy	46 (92%)	32 (97%)	14 (82%)	
Missing	1	1	0	
Estimated Blood Loss (l)	2.00 (1.00, 4.50)	1.85 (1.00, 4.50)	2.00 (1.50, 6.00)	0.3
Received Red Cell Transfusion (%)	41 (80%)	28 (82%)	13 (76%)	0.7
Intravenous crystalloids (l)	2.00 (1.40, 3.00)	2.00 (1.40, 2.80)	2.10 (1.50, 3.00)	0.7
PTX-3 concentration: T4 (ng/mL)	15.9 (8.63, 25.6)	12.4 (6.85, 25.1)	20.1 (13.0, 25.3)	0.12
Delta-PTX-3: T3 to T4 (ng/mL)	7.56 (3.64, 14.0)	6.59 (2.87, 13.2)	12.3 (6.65, 18.7)	0.048

BMI (Body Mass Index); DCD (Donation after Circulatory Death);EAD (Early Allograft Dysfunction); HCC (Hepatocellular Carcinoma); LDLT (Living Donor Liver Transplant); MASH (Metabolic-dysfunction Associated Steatohepatitis); MELD-Na (Model for End-Stage Liver Disease-Sodium); NMP (Normothermic Machine Perfusion); PTX-3 (Pentraxin-3); SCS (Static Cold Storage); WIT (Warm Ischemic Time).

a Median (IQR); *n* (%).

b Wilcoxon rank sum test; Fisher's exact test; Fisher's Exact Test for Count Data with simulated *p*-value (based on 2000 replicates); Pearson's Chi-squared test; Wilcoxon rank sum exact test; Mood's median test.

**Figure 4:**
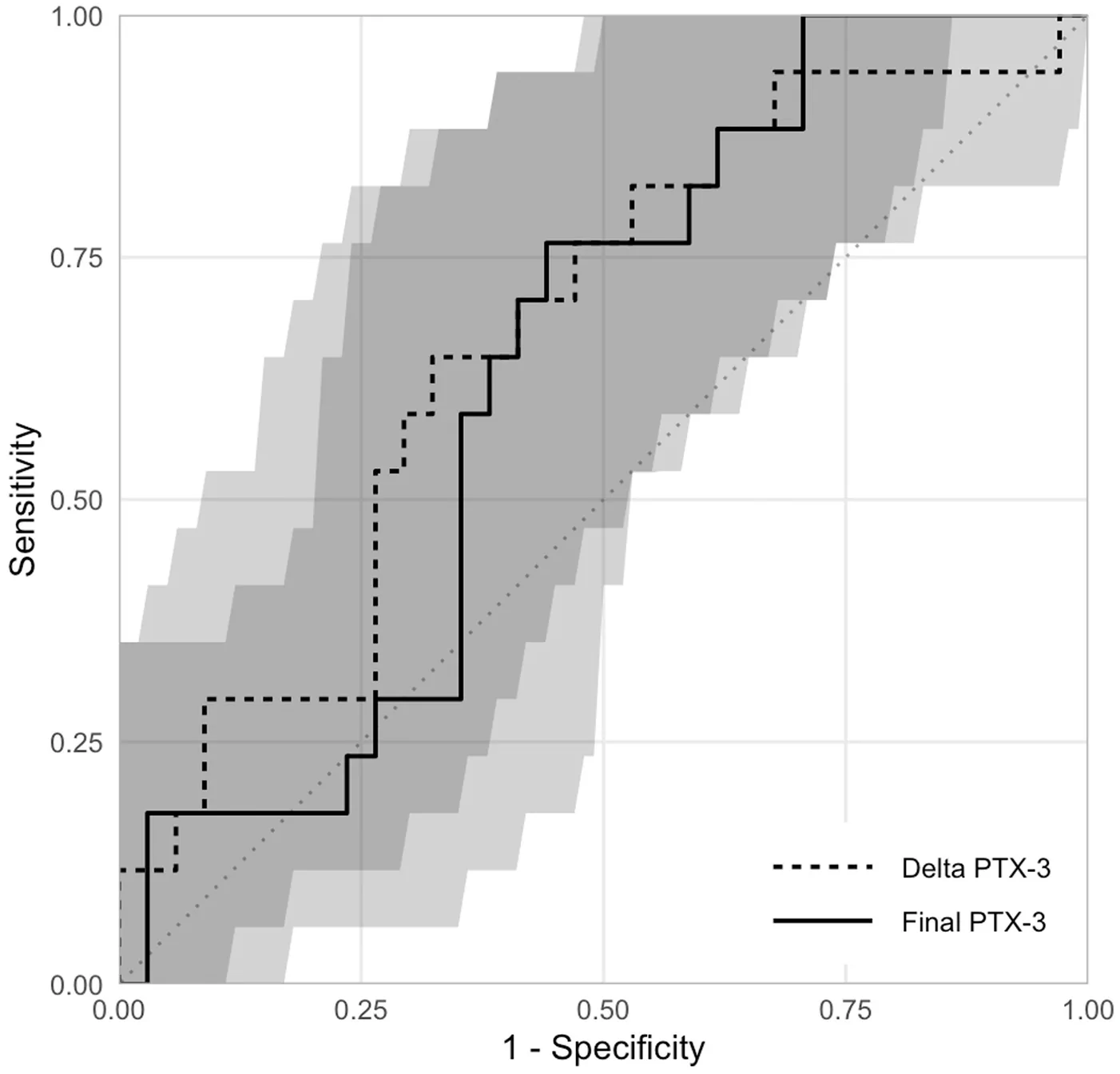
Final (T4) and delta-PTX-3 (T3 to T4) ROC curves for EAD with 95% confidence intervals calculated by stratified bootstrapped resampling (2000 replicates). Final PTX-3 AUROC 0.63 (0.48-0.78); delta-PTX-3 0.67 (0.51-0.82).

The median MEAF score was 4.38 (IQR 3.52–5.72) in those with T4 (*n* = 51), and 4.47 (IQR 3.43–5.64) in all 76 patients. There was no association between MEAF score and the final T4 PTX-3 concentration in a univariate linear regression (*p* = 0.87), nor with delta-PTX-3 T3 to T4 (*p* = 0.21), nor with delta-PTX-3 from the start of surgery, T1 to T4 (*p* = 0.84). AKI occurred within 48 h of surgery in 34 of 51 patients with T4 (67%) and in 52 of all patients (68.4%). There was no significant difference in PTX-3 between those who did and did not develop AKI at T4 or delta-PTX-3 T3 to T4, as provided in Supplementary Table S1. Fifty-nine patients were living without retransplant at least 90 days after surgery at the time of last follow-up. During this time, two patients developed graft failure and had re-transplantation, and one patient died. Fourteen patients were censored as they had fewer than 90 days of follow-up.

## 4 Discussion

We found an increase in circulating PTX-3 during liver transplant surgery, with the highest concentrations after reperfusion, and the change after reperfusion was more than double the change from the start of surgery to reperfusion. In addition, we observed a change in PTX-3 that was an order of magnitude greater in liver transplant recipients than in healthy living donors undergoing living donor surgery. As IRI of the liver graft is a key physiological event distinguishing the later stages of these two surgeries, both of which involve significant surgical tissue trauma, we speculate that IRI of the liver graft may trigger the increase in PTX-3 during liver transplantation (33). Our findings are consistent with a transcriptional study that demonstrated a significant increase in PTX-3 mRNA expression in the hours after reperfusion in human liver biopsies and an association between higher PTX-3 mRNA levels and greater hepatic pathological damage in a mouse model of IRI (20). The same study also demonstrated that siRNA-mediated PTX-3 knockdown in immortalized mouse macrophages suppressed M1 polarization in a hypoxia-reoxygenation model, suggesting a mechanism by which PTX-3 enhances leukocyte-mediated inflammation.

Although we did not observe a difference in peak PTX-3 between those who developed EAD and those who did not, we did observe a difference in delta-PTX-3 after reperfusion. The higher delta-PTX-3 after reperfusion (T3-T4) suggests that reperfusion may activate cells in the donor liver, including endothelial cells and leukocytes (lymphocytes and Kupffer cells). Alternatively, substances flushed from the liver during reperfusion may also induce systemic production or release of PTX-3. We speculate that reperfusion-associated increases in PTX-3 levels induce endothelial dysfunction, which, in turn, contributes to IRI-induced allograft dysfunction. This is supported by *in vitro* evidence that PTX-3 induces vascular dysfunction through *P*-selectin and Matrix Metalloproteinase-1 signaling (34). This concept is consistent with positive responses to interventions targeting endothelial dysfunction, such as *P*-selectin-blocking agents and statins, which have shown promise in early clinical trials (35, 36).

In contrast to these results showing a relationship between delta-PTX-3 and EAD, we did not observe a relationship between delta-PTX-3 and MEAF score, which measures allograft dysfunction on a continuous scale. This differing relationship may reflect differences in how biomarkers are handled within the algorithms used for these measures. MEAF uses inverse natural log-transformed biomarkers, whereas EAD (Olthoff) uses binary thresholds, resulting in different weights for biomarker components in each score.

Donor, recipient, and surgical factors predictive of EAD are reported to include recipient MELD score, and donor type (8, 37, 38). We found no significant differences in MELD-Na by EAD (Table 3). Recipients who developed EAD differed by donor and graft preservation type. All those with T4 with grafts from DCD had EAD, although delta-PTX-3 did not differ by DCD. However, the association between delta-PTX-3 and EAD may still be affected by confounding by DCD status.

A novel finding of our study was the unexpected relationship between graft preservation type and delta-PTX-3. Specifically, delta-PTX-3 levels were significantly higher in those who received grafts that underwent NMP than in those who underwent LDLT, but the difference in absolute T4 PTX-3 values was not significant. Both PTX-3 final and delta-PTX-3 trended higher in grafts with NMP than those that underwent SCS, but these differences did not reach significance after correction for multiple comparisons. We speculate that NMP may induce PTX-3 earlier in time than in SCS or LDLT donor endothelial cells or leukocytes, as other studies have reported increased endothelial activation during NMP (39). PTX-3 expression was reported to increase in bulk RNA sequencing of liver graft biopsies before and after normothermic perfusion, ranking among the top 40 differentially expressed genes (40). This may also reflect an increased proportion of higher-risk grafts in NMP cohorts, as evidenced by changing risk profiles of liver transplants nationally (41). We observed similar PTX-3 levels in DCD cases that underwent NMP or SCS, although the sample size was very small. However, we also observed similar Donor Risk Index and proportions of grafts from DCD in both groups that underwent NMP and SCS.

We observed an association between the MELD-Na score and initial PTX-3 concentration, consistent with PTX-3's role as a marker of inflammation and the proinflammatory state observed in patients with high MELD-Na scores (11, 42). This relationship was not observed at later timepoints. Additionally, the PTX-3 range among patients at the start of surgery (IQR: 1.15–2.95 ng/mL) was considerably less than that observed after reperfusion (IQR: 8.38–25.1 ng/mL). This suggests IRI and the changes around transplant were the greater drivers of delta-PTX-3 compared to preoperative inflammation. Although PTX-3 expression in tumors is a marker of poor prognosis in HCC, we did not find an association between the initial circulating PTX-3 concentration and HCC (43).

In our study, PTX-3 was higher in males versus females at both the start of surgery and 60–120 min after reperfusion. In contrast, baseline circulating PTX-3 levels have been reported to be higher in females (15). The relationship between sex and PTX-3 in our study may have been influenced by the overrepresentation of females in the graft preservation type groups, rather than a different relationship between sex and PTX-3 in pathological states than that in healthy populations.

The volume of crystalloids given intraoperatively was positively associated with higher final PTX-3, even though we might have expected a dilutional effect to lower PTX-3. Given the observed increase in PTX-3 despite the infused volume, this further supports the significant change in PTX-3 concentration that we observed following reperfusion.

This study has several strengths and novel findings. Firstly, reporting the measurement of the biomarker PTX-3 over the course of liver transplant in relation to clinically significant outcomes. Secondly, this work has generated results that suggest further hypotheses for investigation, such as the novel finding that delta-PTX-3 levels, but not final PTX-3 levels, were higher in those with normothermic machine-perfused livers. Thus further investigation is warranted into the association between machine perfused livers and drivers of endothelial activation, the components and timing of such activation, and associated clinical risk profiles and outcomes. Thirdly, the cohort design enabled the description of PTX-3 in relation to multiple outcomes and exposures.

There are a few limitations to our study. Firstly, this was a retrospective cohort study with a limited sample size, including high proportions of LDLT and NMP, which precluded further adjusted stratified analysis by graft preservation and donation type. The AUROC for delta-PTX-3 indicated low discrimination, and we did not compare to classical biomarkers of endothelial dysfunction (44). The sequential paired data sample size was also limited by the availability of research staff during out-of-hours transplant surgery. Nevertheless, these findings have generated hypotheses for further investigation. Future work should examine the relationship between components of endothelial activation after IRI and normothermic machine perfusion of grafts and how this relates to clinical outcomes.

Our data indicate that PTX-3 levels increase over the course of liver transplantation, particularly after reperfusion. This suggests that IRI drives the rise in PTX-3. We speculate that PTX-3 promotes endothelial activation and dysfunction in transplanted livers, but further work is needed to clarify its effects on the liver endothelium, the graft inflammatory microenvironment, and liver transplantation outcomes. Similarly, additional studies are needed to understand the role of the endothelium in IRI and EAD, and whether endothelial dysfunction mediates clinical outcomes of liver transplantation. A better understanding of PTX-3's complex role may help elucidate the pathophysiological mechanisms underlying patients' disease and identify targets for developing future therapies to improve liver transplantation outcomes.

## Data Availability

The raw data supporting the conclusions of this article will be made available by the authors, without undue reservation.
