# Supplementary material for: Pentraxin-3 dynamics during liver transplantation and association with early allograft dysfunction
Source: Front Transplant. 2026 Oct 16;5:1920891. doi: 10.3389/frtra.2026.1920891 (PMC13719452; doi:10.3389/frtra.2026.1920891)
Supplement: Supplementary file 1 [file Table1.docx]

**Supplement Table 1:** Change in Pentraxin-3 concentration after reperfusion and final Pentraxin-3 concentration by Acute Kidney Injury status

|  | **Delta-PTX-3 (T3 to T4)** | |  | **PTX-3 at T4** | |  |
| --- | --- | --- | --- | --- | --- | --- |
| **Group** | **Median ng/mL (IQR)** | **p value^1^** | **n** | **Median ng/mL (IQR)** | **p value^1^** | **n** |
| All recipients | 7.88 (3.69-14.1) |  |  | 16.7 (9.18-26.4) |  |  |
| **AKI Comparison** |  |  |  |  |  |  |
| AKI absent | 7.40 (3.37-17.7) | p=0.81 | 29 | 12.6 (7.38-27.7) | p=0.08 | 29 |
| AKI present | 7.88 (4.23-13.9) |  | 16 | 19.6 (11.4-25.8) |  | 16 |

*^1^*p values from the Wilcoxon rank-sum test and Mood’s median test

AKI (Acute Kidney Injury); IQR (Interquartile Range); PTX-3 (Pentraxin-3)
